# TRPM4 regulates Akt/GSK3‐β activity and enhances β‐catenin signaling and cell proliferation in prostate cancer cells

**DOI:** 10.1002/1878-0261.12100

**Published:** 2017-12-30

**Authors:** Alfredo I. Sagredo, Eduardo A. Sagredo, Claudio Cappelli, Pablo Báez, Rodrigo E. Andaur, Constanza Blanco, Julio C. Tapia, César Echeverría, Oscar Cerda, Andrés Stutzin, Felipe Simon, Katherine Marcelain, Ricardo Armisén

**Affiliations:** ^1^ Centro de Investigación y Tratamiento del Cáncer Facultad de Medicina Universidad de Chile Santiago Chile; ^2^ Instituto de Ciencias Biomédicas Facultad de Medicina Universidad de Chile Santiago Chile; ^3^ Departamento de Oncologia Basico‐Clinica Facultad de Medicina Universidad de Chile Santiago Chile; ^4^ Programa de Biología Celular y Molecular ICBM Facultad de Medicina Universidad de Chile Santiago Chile; ^5^ Cell Transformation Laboratory Facultad de Medicina Universidad de Chile Santiago Chile; ^6^ Centro Integrativo de Biología y Química Aplicada Universidad Bernardo OHiggins Santiago Chile; ^7^ Laboratorio de Fisiopatologia Integrativa Departamento de Ciencias Biologicas Facultad de Ciencias Biologicas and Facultad de Medicina Universidad Andres Bello Santiago Chile; ^8^ Millennium Institute on Immunology and Immunotherapy Santiago Chile; ^9^ Present address: Center of Excellence in Precision Medicine Pfizer Chile Obispo Arturo Espinoza Campos 2526, Macul 7810305 Santiago Chile

**Keywords:** ion channel, prostate cancer, TRPM4, Wnt, β‐catenin

## Abstract

Increased expression of the TRPM4 channel has been reported to be associated with the progression of prostate cancer. However, the molecular mechanism underlying its effect remains unknown. This work found that decreasing TRPM4 levels leads to the reduced proliferation of PC3 cells. This effect was associated with a decrease in total β‐catenin protein levels and its nuclear localization, and a significant reduction in Tcf/Lef transcriptional activity. Moreover, TRPM4 silencing increases the Ser33/Ser37/Thr41 β‐catenin phosphorylated population and reduces the phosphorylation of GSK‐3β at Ser9, suggesting an increase in β‐catenin degradation as the underlying mechanism. Conversely, TRPM4 overexpression in LNCaP cells increases the Ser9 inhibitory phosphorylation of GSK‐3β and the total levels of β‐catenin and its nonphosphorylated form. Finally, PC3 cells with reduced levels of TRPM4 showed a decrease in basal and stimulated phosphoactivation of Akt1, which is likely responsible for the decrease in GSK‐3β activity in these cells. Our results also suggest that the effect of TRPM4 on Akt1 is probably mediated by an alteration in the calcium/calmodulin‐EGFR axis, linking TRPM4 activity with the observed effects in β‐catenin‐related signaling pathways. These results suggest a role for TRPM4 channels in β‐catenin oncogene signaling and underlying mechanisms, highlighting this ion channel as a new potential target for future therapies in prostate cancer.

**Abbreviations**
CaM: calmodulin
EGF: epidermal growth factor
EGFR: epidermal growth factor receptor
GSK‐3β: glycogen synthase kinase 3β
LNCaP: lymph node carcinoma of the prostate
PC3: prostatic carcinoma cell line 3
ShTRPM4: short hairpin RNA against TRPM4 mRNA
TCF/LEF: T‐cell factor‐1/lymphoid enhancing factor 1
TRPM4: transient receptor potential cation channel subfamily M member 4
Wnt: Wingless‐Int

## 1 Introduction

Prostate cancer is one of the most frequently diagnosed malignancies and the fifth cause of cancer‐related deaths in men (Torre *et al*., 2015). Although the clinical and the histopathological features of these tumors are well defined, the genetic and molecular changes during the progression of prostate cancer are poorly understood (Schrecengost and Knudsen, 2013; Tindall, 2013). Prostate tumors are characterized by multiple foci of origin (Schrecengost and Knudsen, 2013), with a large number of genetic alterations. Some genetic abnormalities frequently found in prostate cancer are the loss of important tumor suppressor genes such as PTEN, NKX3.1, TP53 (Mazaris and Tsiotras, 2013; Shen and Abate‐Shen, 2010) and the formation of oncogenic fusions such as TMPRSS2‐ERG (King *et al*., 2009; Tomlins *et al*., 2005). Also, the activation of several signaling pathways including Wnt/β‐catenin (Kypta and Waxman, 2012; Wang *et al*., 2008) has been shown to contribute to the development and progression of this cancer.

In normal nonstimulated cells, β‐catenin, the main effector of the canonical Wnt pathway (Valenta *et al*., 2012), is localized at adhesion complexes in the cell membrane, while the cytoplasmic protein levels of β‐catenin are tightly regulated (MacDonald *et al*., 2009) by a multiprotein structure known as the destruction complex. This allows the phosphorylation of β‐catenin by GSK‐3β at specific Ser/Thr residues (Ser33/Ser37/Thr41) (Stamos and Weis, 2013), which promotes its proteasome‐mediated degradation, circumventing its nuclear translocation and ulterior transcriptional function (MacDonald *et al*., 2009). The stimulation of the Wnt/canonical pathway by specific ligands (Rao and Kühl, 2010), activating mutations on the β‐catenin sequence or inactivation of GSK‐3β by specific phosphorylation at Ser9, stabilizes β‐catenin. This promotes its translocation into the nucleus and interaction with members of the Tcf/Lef family of transcription factors (Mosimann *et al*., 2009), leading to the expression of genes involved in proliferation, apoptosis, and invasion, among other important processes for cancer progression (Clevers, 2006; Klaus and Birchmeier, 2008). Moreover, in prostate cancer, increased β‐catenin levels and nuclear localization correlate with the progression of the disease (de la Taille *et al*., 2003; Whitaker *et al*., 2008), suggesting a possible alteration in the regulation of this protein. However, as activating mutations on β‐catenin are not common in prostate cancer, the mechanisms of this misregulation are not yet clear (Kypta and Waxman, 2012).

TRPM4, a calcium‐activated monovalent‐selective cation channel (Guinamard *et al*., 2010; Launay *et al*., 2002), has been found to be overexpressed in a variety of human tumors, including prostate cancer (Berg *et al*., 2016; Holzmann *et al*., 2015; Prevarskaya *et al*., 2007; Suguro *et al*., 2006). TRPM4 expression is increased in the transition from prostatic intraepithelial neoplasia (PIN) to prostate cancer (Ashida *et al*., 2004; Singh *et al*., 2006). This channel controls the frequency and magnitude of Ca^2+^ influx by modulating the membrane potential and subsequently the driving force for Ca^2+^ influx through other Ca^2+^‐permeable pathways (Fliegert *et al*., 2007; Launay *et al*., 2004; Nilius and Vennekens, 2006; Weber *et al*., 2010). Holzmann *et al*. (2015) showed TRPM4‐like currents in prostate cancer cell lines and it has a role as a negative feedback for Ca^2+^ entry. Considering intracellular calcium's extensive role as a second messenger involved in many of the signaling pathways responsible for cancer progression (Farfariello *et al*., 2015; Monteith *et al*., 2007), the identification of specific pathways that have been altered after an aberrant change in intracellular calcium signals has been very challenging. Also, the overexpression of TRPM4 has been shown to promote the stabilization and activity of β‐catenin enhancing cell proliferation in HeLa cells, but the underlying mechanisms have not yet been clarified (Armisén *et al*., 2011).

This study presents further evidence to show that TRPM4 regulates β‐catenin signaling and enhances the proliferation of prostate cancer cell lines, through a calcium‐dependent regulation of Akt1 and GSK‐3β activity. TRPM4 silencing resulted in a reduced proliferation of PC3 cells. In these cells, diminished levels of TRPM4 channels resulted in a decrease in total and nuclear β‐catenin protein levels and its transcriptional activity, while the Ser33/Ser37/Thr41 phosphorylated fraction was increased. Also, these results correlated with a reduction of phospho‐Ser9 on GSK‐3β, indicating an increase in this enzyme activity. Moreover, the overexpression of TRPM4 in LNCaP cells increased the total levels of β‐catenin and the inhibitory phosphorylation of GSK‐3β. Finally, the knockdown of TRPM4 correlated with a decrease in basal and stimulated phosphoactivation of Akt1, a well‐known GSK‐3β regulator, by altering the calcium/calmodulin‐EGFR axis, and linking the TRPM4 channel expression to the control of cellular proliferation.

## 2 Materials and methods

### 2.1 Cell culture

The human prostate cancer cells PC3 and LNCaP were kept in RPMI 1640 (Corning Inc, Corning, NY, USA). T‐REx‐293‐TRPM4 and HEK293 cells were kept in DMEM‐low glucose (Corning Inc). All the growth media were supplemented with 10% v/v FBS (Corning) and penicillin/streptomycin (Hyclone Laboratories, Logan, UT, USA). RWPE‐1 cells were kept in keratinocyte‐SFM (Thermo Scientific, Waltham, MA, USA) supplemented with recombinant human epidermal growth factor (K‐SFM kit) and bovine pituitary extract (K‐SFM kit).

### 2.2 Antibodies

The following antibodies were used in this study: mouse anti‐TRPM4b (Origene, Rockville, MO, USA, TA500381), mouse anti‐β‐catenin (610154; BD Biosciences, San Jose, CA, USA), mouse anti‐non‐phosphorylated S33/S37/T41‐β‐catenin (Millipore, Temecula, CA, USA, 05‐665), rabbit anti‐phospho‐Ser33/Ser37/Thr41 β‐catenin (Cell Signaling Technology, Danvers, MA, USA, 9561), mouse anti‐GSK‐3β (BD Biosciences, 610201), rabbit anti‐phospho‐Ser9 GSK‐3β (Origene, Rockville, MO, USA, TA303847), rabbit anti‐Akt1 (Cell Signaling, 9272), rabbit anti‐phospho‐Ser473 Akt (Cell Signaling, 9271), and as loading controls, mouse anti‐α‐tubulin (Sigma Aldrich, St. Louis, MO, USA, T5168) or mouse anti‐HSP70 (Origene, TA309356).

### 2.3 Drugs and recombinant protein

For the activation of Akt, epidermal growth factor recombinant protein (R&D Systems, Minneapolis, MN, USA, 236‐EG) was added to the growth media (EGF, 100 ng·mL^−1^/15 min). 9‐Phenanthrol (Sigma‐Aldrich, 211281) at 10 μm final concentration in the growth media for 2 h was used for the inhibition of TRPM4. DMSO was used as a vehicle. Before the experiments, tetracycline (Sigma‐Aldrich, T7660) was added to the growth media for TRPM4 induction at a final concentration of 1 μg·mL^−1^ for 24 h. The calmodulin inhibitor W‐7 (Tocris Bioscience, Bristol, UK, 0369) was used at a final concentration of 100 μm for 1 h before the incubation with EGF.

### 2.4 Transfection and transductions

PC3 cells were transduced with a commercial prepackaged lentiviral vector (SBI, Palo Alto, CA, USA) directing shRNA against TRPM4 mRNA (ShTRPM4) or a scramble ShRNA (ShControl) as a control. Cells were kept in growth media with 0.8 μg·mL^−1^ puromycin (Corning) for selection. LNCaP cells were transfected with pcDNA4TO/TRPM4b (human) plasmid or an empty vector (mock), using Lipofectamine LTX with Plus Reagent (Invitrogen, Carlsbad, CA, USA), and held in growth media with 50 μg·mL^−1^ zeocin (Corning) for selection.

### 2.5 Immunoblotting

Protein lysates were prepared in a RIPA buffer (25 mm Tris/HCl pH 7.6, 150 mm NaCl, 5 mm EDTA, 1% v/v Triton X‐100, 1% w/v sodium deoxycholate, 0.1% w/v SDS) and a protease (Calbiochem, San Diego, CA, USA) and phosphatase (Roche Life Sciences, Mannheim, Germany) inhibitor cocktail. Protein lysates (30 μg per lane) were resolved on 8% sodium dodecyl sulfate/polyacrylamide gel electrophoresis (SDS/PAGE), and proteins were transferred onto a nitrocellulose membrane. Membranes were blocked in 5% w/v BSA (Winkler, Santiago, Chile) and then incubated with primary antibodies at 4 °C overnight. All primary antibodies were detected using appropriate HRP‐conjugated secondary antibodies and a chemiluminescence reagent (SuperSignal WestPico Chemiluminescent Substrate; Thermo Scientific), and images were obtained using the ChemiScope3500 Mini chemiluminescence imaging system (Clinx Science Instruments, Shanghai, China).

### 2.6 Quantitative PCR

Total RNA was extracted using TRIzol (Invitrogen), followed by DNase treatment (TURBO DNase; Ambion, Austin, TX, USA). One microgram of RNA was reverse‐transcribed using the AffinityScript qRT‐PCR cDNA Synthesis Kit (Agilent Technologies, Inc., Santa Clara, CA, USA) and diluted five times. Quantitative expression analysis was performed using specific oligonucleotide primers and Brilliant II SYBR Green qRT‐PCR Master Mix (Agilent). The reactions were quantified with an Eco Real‐Time (Illumina, San Diego, CA, USA) using the following program: 95 °C for 15 s, 58 °C for 15 s, and 72 °C for 15 s at 40 cycles. Expression values were calculated using the ΔΔ*C*_t_ method and expressed as the fold change relative to control samples. GAPDH was used as a housekeeping gene. The primers sequences (5′→3′) are as follows:

GAPDH (Fw GTTGCTGTAGCCAAAT TCGTTGT, Rv GGTGGTCTCCTCTGACTTCAACA),
BIRC5 (Fw GCTTCGCTGGAAACCTCTGGA, Rv TCTGGGCAGATGGCTGTTGG),
MYC (Fw ACAGCCCACTGGTCCTCAAGA, Rv ACCTGGGGCTGG TGCATTTT),
CCND1 (Fw CACTTGCATGTTCGTGGCCTCTA, Rv ATTGCGGCCAGGTTCCACTT),
TRPM4 (Fw TCGGCAAAGTACAGGGCAAC, Rv AGGCGCAAGTGGGAGATGAC)
AXIN2 (Fw AGCCAAAGAAACTGGCAGGTGT, Rv GTCAAGCTCTGAGCCTTCAGC).

### 2.7 Indirect immunofluorescence

Cells were fixed (4% w/v formaldehyde; Sigma‐Aldrich), permeabilized, blocked, and incubated with anti‐β‐catenin (1 : 250) followed by anti‐mouse coupled with Alexa‐Fluor546 (Invitrogen, 1 : 1000). Cells were stained and mounted in Prolong‐DAPI (Invitrogen). Images were recorded in an inverted microscope (IX81 Spinning Disk Confocal; Olympus, Center Valley, PA, USA) and analyzed using imagej software (Rasband, 2015) to measure the relative levels of nuclear and cytoplasmic β‐catenin fluorescence.

### 2.8 TOP/FOP luciferase reporter assay

Cells were transiently transfected with 0.5 μg of constitutively active vector encoding Renilla luciferase (Promega, Madison, WI, USA) and 2 μg of β‐catenin responsive firefly luciferase reporter plasmid TopFlash or the negative control FopFlash (Merck Millipore, Billerica, MA, USA). Cells were harvested 24 h after transfection and firefly and Renilla luciferase activities were measured in triplicate using the dual luciferase kit (Dual‐Glo Luciferase Assay System; Promega). The firefly luciferase activity was normalized against the Renilla luciferase activity, following Armisén *et al*. (2011).

### 2.9 Calcium measurements

Cells were seeded on 25‐mm glass coverslips. At 70% confluence, growth media were replaced with an extracellular buffer with calcium (ECM + Ca^2+^: 145 mm NaCl, 5 mm KCl, 1 mm MgCl_2_, 1 mm CaCl_2_, 10 mm HEPES, pH 7.4), and the cells were loaded with Fura‐2 (2 μm; Molecular Probes, Eugene, OR, USA) for 1 h at 37 **°**C. Then, the ECM + Ca^2+^ solution was removed and cells were washed twice with calcium‐free ECM (plus 5 mm EGTA). After baseline recording, the SERCA inhibitor thapsigargin (1 μm) was perfused. After a transient increase in cytosolic calcium concentration [denoted as endoplasmic reticulum (ER) calcium leak], the calcium‐free external solution was replaced with ECM + Ca^2+^ buffer to record calcium influx (after stored depletion). The ratiometric fluorescence (R_340/380_) of the Fura‐2 calcium indicator was measured in an inverted microscope (IX81 Spinning Disk Confocal; Olympus) and recorded and analyzed by the cellr imaging software (Olympus) as described in Echeverría *et al*. (2014).

### 2.10 MTS assay

Cells were seeded in 24‐well plates (10 × 10^3^ cells/well) and allowed to attach overnight. Cell proliferation was assessed using the Cell‐Titter 96 AQueous‐MTS Kit (Promega) at 24, 48, and 72 h. Absorbance was measured at 490 nm in the Cytation 3 Multi‐Mode Reader (Biotek Instrument, Winooski, VT, USA).

### 2.11 DNA synthesis/BrdU Incorporation

DNA synthesis evaluated by bromodeoxyuridine (BrdU)/propidium iodide (PI) staining was as follows: exponentially growing cells were incubated with 25 μm of BrdU (Santa Cruz Biotechnology, Santa Cruz, CA, USA) for 45 min before harvesting. Cells were fixed in 80% methanol at −20 °C and kept overnight at the same temperature. Double staining with 50 μg·mL^−1^ PI and FITC‐anti‐BrdU antibody (BD Biosciences Pharmingen, San Diego, CA, USA) was performed according to the manufacturer's protocol. Cell cycle profiles and BrdU uptake were determined by FACS (BD Bioscience), and the data were analyzed with the bd facsdiva software (BD Bioscience).

### 2.12 Statistical analysis

qRT‐PCR, western blot, and functional assays were examined by Student's *t*‐test with Welch correction or the ANOVA test, whichever is applicable. *P* < 0.05 was considered statistically significant. At least three independent experiments were performed for each analysis. Statistical analysis was performed using prism 5.0 (GraphPad Software, San Diego, CA, USA). Material and Methods for supplemental results can be found in Data S1.

## 3 Results

### 3.1 TRPM4 regulates β‐catenin activity and cell proliferation in prostate cancer cells

**Figure 1:**
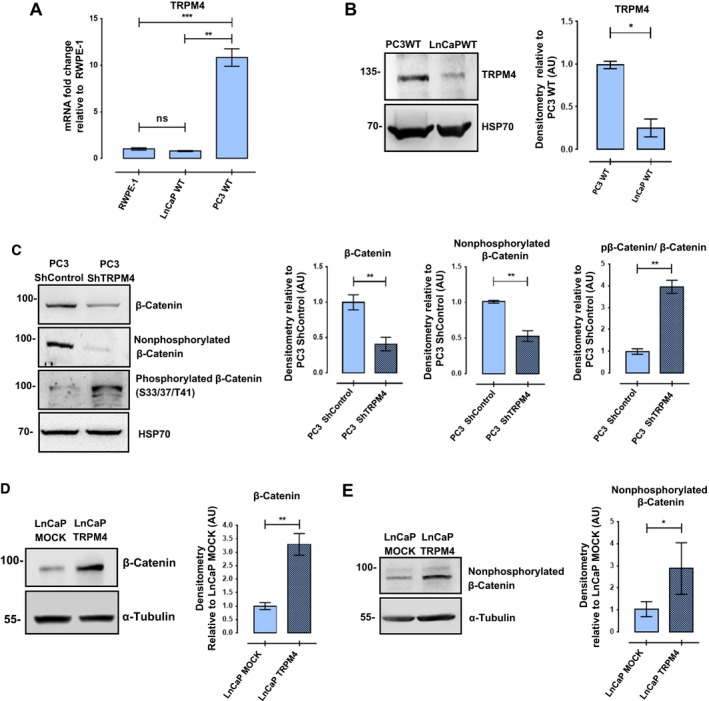
TRPM4 expression affects protein levels and phosphorylation status of β‐catenin. (A) mRNA expression of TRPM4 gene in PC3 WT and LNCaP WT cells relative to nontumoral RWPE‐1 prostate cells. RT‐qPCR were performed at least three times. *T‐*test with Welch correction, ^ns^ *P* > 0.05, **P ≤ 0.01, ***P ≤ 0.001. (B) Western blot was performed to detect TRPM4 expression in prostate cancer cell lines. Representative western blots and their densitometries from three independent experiments. Mean ± SEM. *T‐*test with Welch correction, *P ≤ 0.05. (C) Knockdown of TRPM4 in PC3 cells resulted in a significant decrease in total β‐catenin protein levels, along with the nonphosphorylated β‐catenin (‘active’), and an increase in phosphorylated β‐catenin at Ser33/37/Thr41 residues. *T‐*test with Welch correction, **P ≤ 0.01. (D, E) TRPM4‐transfected LNCaP cells show an increase in total and nonphosphorylated β‐catenin protein levels compared to MOCK‐transfected cells. *T‐*test with Welch correction, *P ≤ 0.05, **P ≤ 0.01. Representative western blots and their densitometries from at least three experiments. Mean ± SEM are shown.

TRPM4 mRNA expression was analyzed in a nontransformed prostate epithelial RWPE‐1 cell line and two cancer‐derived cell lines, LNCaP and PC3. As shown in Fig. 1A, PC3 cells express 10‐fold more TRPM4 mRNA than LNCaP and RWPE‐1 cells. At the protein level, LNCaP cells showed a significantly lower TRPM4 expression than PC3 cells (Fig. 1B). To assess the role of TRPM4 in prostate cancer, knockdown and overexpression of TRPM4 were performed in PC3 and LNCaP cells, respectively. Immunoblot and qPCR analysis were used to determine the knockdown efficiency and possible off‐target effects (Figs S1 and S2).

**Figure 2:**
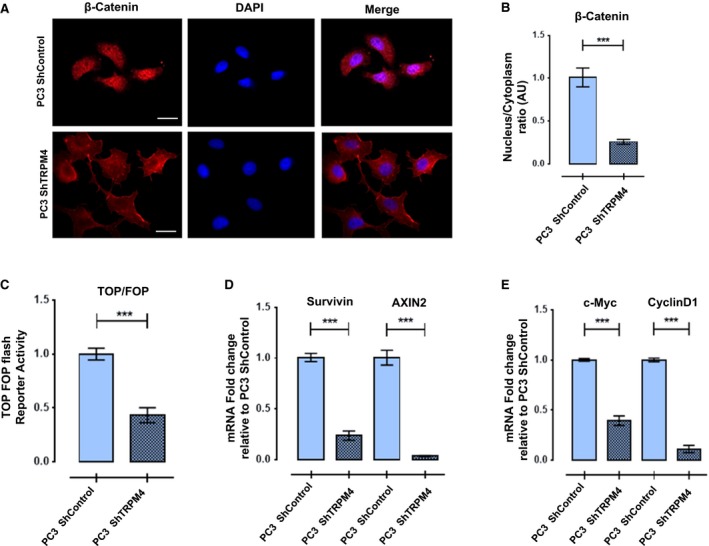
TRPM4 expression effects, nuclear distribution, and transcriptional activity of β‐catenin. (A, B) Knockdown of TRPM4 in PC3 cells decreases β‐catenin nuclear localization. Representative images of intracellular β‐catenin detected by immunofluorescence in PC3 ShControl (upper) and PC3 ShTRPM4 (bottom). Scale bar 20 μm. Graph shows arbitrary units of fluorescence of β‐catenin nuclear/cytoplasmic ratio quantified in at least three independent experiments. Mean ± SEM are shown. *T‐*test with Welch correction, ***P ≤ 0.001. (C) Knockdown of TRPM4 in PC3 cells decreases β‐catenin cotranscriptional activity. PC3 ShTRPM4 and PC3 ShControl cells were transfected with plasmid pTOP or pFOP and pTK‐Renilla as normalizer. Normalized TOP/FOP Luciferase activity is shown. *T‐*test with Welch correction, ***P ≤ 0.001. (D, E) Relative mRNA expression of β‐catenin target genes. Axin2, survivin, c‐Myc, and cyclin D1 genes were analyzed in PC3 ShTRPM4 compared to PC3 ShControl cells with RT‐qPCR assay. All experiments were performed at least three times. Mean ± SEM are shown*. T‐*test with Welch correction, ***P ≤ 0.001.

**Figure 3:**
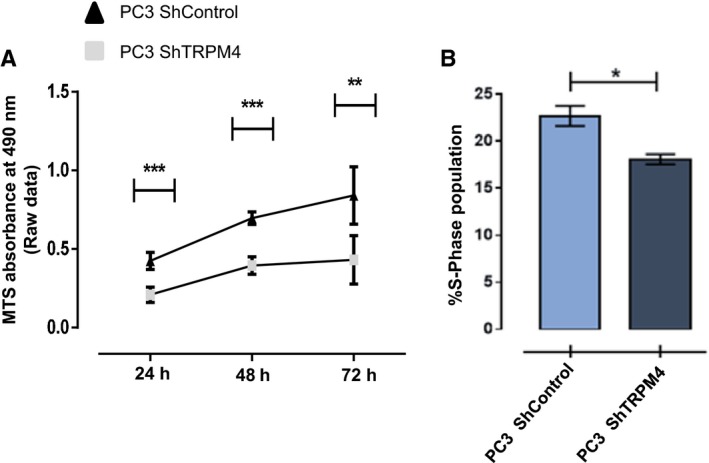
TRPM4 knockdown reduces proliferation of PC3 cells. (A) Cell proliferation was evaluated in PC3 ShTRPM4 and PC3 ShControl cells, by MTS assay. Three independent assays were performed, in triplicate, and proliferation was assessed 24–72 h after culturing. Mean ± SEM are shown. Two‐way ANOVA, **P ≤ 0.005, ***P ≤ 0.001. (B) PC3 ShTRPM4 and ShControl cells were pulsed with BrdU for 45 min under normal culturing conditions. Positive cells that incorporated BrdU were detected using a FITC‐conjugated anti‐BrdU antibody. Cells in S‐phase were selected according to DNA content (propidium iodide signal). All experiments were performed in at least three independent experiments. Mean ± SEM are shown. Mann–Whitney *test*, *P ≤ 0.05.

Previous work showed a positive relationship between the expression of TRPM4 channel and total β‐catenin protein levels in HeLa cells (Armisén *et al*., 2011); therefore, the effect of TRPM4 knockdown on β‐catenin was explored in PC3 prostate cells. As expected, a significant decrease in the total amount of β‐catenin was found in PC3 ShTRPM4 cells compared to PC3 ShControl cells. The nonphosphorylated fraction of β‐catenin (Ser37 and/or Thr41) also decreased, while the phosphorylated population (Ser33, Thr37 or 41) increased (Fig. 1C), suggesting a rise in β‐catenin degradation in PC3 ShTRPM4 cells. Conversely, LNCaP cells overexpressing TRPM4 showed a significant increase in total β‐catenin (Fig. 1D) and in the nonphosphorylated fraction, compared to mock‐transfected cells (Fig. 1E). As depicted in Fig. 2A,B, PC3 ShTRPM4 cells also showed a significant decrease in nuclear β‐catenin. Consequently, PC3 ShTRPM4 cells display a lower specific Tcf/Lef transcriptional reporter activity compared to control cells (Fig. 2C), suggesting an alteration in the β‐catenin transcription co‐activator activity. To examine further the effect of TRPM4 knockdown and β‐catenin transcriptional activity, the expression of survivin, axin2, cyclin D1, and c‐Myc by RT‐qPCR was analyzed. PC3 ShTRPM4 cells showed a significant decrease in all these β‐catenin target genes (Niehrs and Acebron, 2012; Tapia *et al*., 2006) (Fig. 2D,E). As c‐Myc and cyclin D1 genes are positive regulators of cell proliferation, we evaluated the effect of TRPM4 knockdown in prostate cancer cell proliferation through MTS and BrdU incorporation assays. These analyses showed that PC3 ShTRPM4 cells have a significantly reduced proliferation (Fig. 3A,B). In order to assess whether the reduced viability observed in PC3 ShTRPM4 cells was a result of an increased cell death, caspase‐3 activation, as well as caspase 3/7 activities, was assessed in PC3 ShTRPM4 and control cells. No significant differences were detected either in total and cleaved caspase‐3 form or in caspase 3/7 activities (Fig. S3). These data suggest that TRPM4 silencing causes a decrease in cellular proliferation.

### 3.2 TRPM4 inhibits GSK‐3β activity

**Figure 4:**
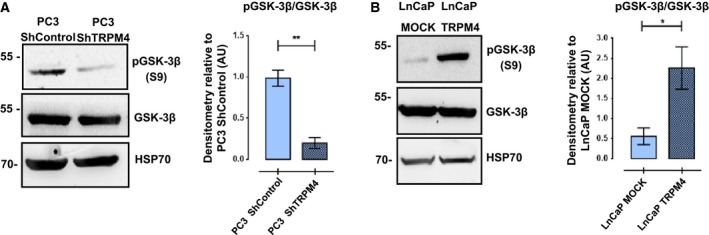
TRPM4 levels are positively related to the inhibitory phosphorylation of GSK‐3β. (A) PC3ShTRPM4 cells show decreased phosphorylation of Ser9 in GSK‐3β, relative to PC3 ShControl cells. No changes in total GSK‐3β were detected. Representative western blots and densitometries of three independent experiments (mean ± SEM) are shown. *T‐*test with Welch correction, **P ≤ 0.01. (B) Overexpression of TRPM4 in LNCaP cells correlated with an increase in phosphorylated Ser9 in GSK‐3β compared to control cells. No changes in the total amount of GSK‐3β were detected. *T‐*test with Welch correction, *P ≤ 0.05.

Given that PC3 ShTRPM4 cells have increased levels of β‐catenin GSK‐3β‐dependent phosphorylated residues, we hypothesized that TRPM4 silencing increases the activity of this kinase. GSK‐3β activity was then assessed by evaluating its inhibitory phosphorylation in Ser9 residue (McManus *et al*., 2005). PC3 ShTRPM4 cells exhibit a significantly lower Ser9 phosphorylation compared to control cells, which is consistent with this enzyme's higher activity (McManus *et al*., 2005). Also, no differences in GSK‐3β total protein levels were observed (Fig. 4A). Subsequently, the inhibitory Ser9 phosphorylation of GSK‐3β was determined in LNCaP cells overexpressing TRPM4. A significant increase in phospho‐Ser9 GSK‐3β in LNCaP‐TRPM4 cells was detected compared to control cells (Fig. 4B), indicating a positive relationship between TRPM4 expression and the status and activity of GSK‐3β/Ser9 phosphorylation.

**Figure 5:**
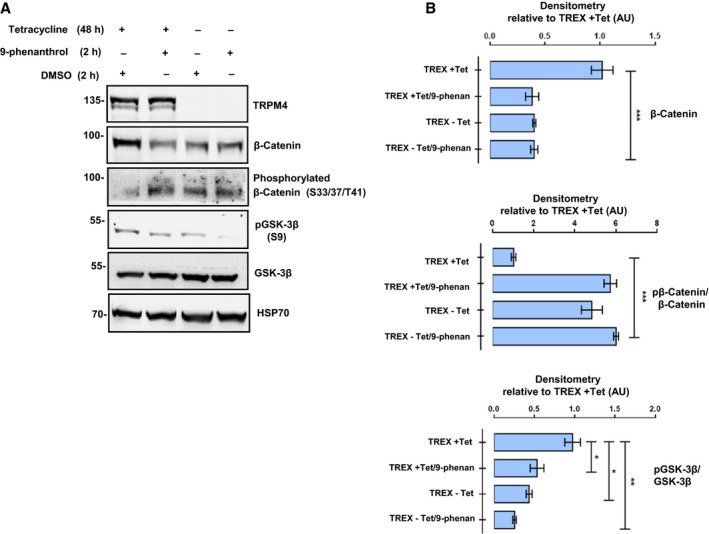
Activity of TRPM4 channel is required for β‐catenin stability and GSK‐3β inhibitory phosphorylation. Overexpression of TRPM4 in T‐REx 293 (TREX) cells was induced by previous incubation with tetracycline 1 μg·mL^−1^ (Tet). Cells were incubated for 2 h with the specific TRPM4 inhibitor 9‐phenanthrol at 10 μm or DMSO (vehicle) before protein extraction. (A, B) TRPM4 overexpression enhances β‐catenin protein levels, decreases its inhibitory phosphorylation, and increases the GSK‐3β S9 phosphorylation. The presence of 9‐phenanthrol reverts the effects of the TRPM4 overexpression on these intracellular proteins. (A) Representative western blots and the densitometries of at least three independent experiments (mean ± SEM) are shown. *P ≤ 0.05; **P ≤ 0.01; ***P ≤ 0.001, multiple *t*‐test comparisons using *t*‐test with Welch correction.

To determine whether TRPM4 activity is required to regulate the status of β‐catenin and GSK‐3β phosphorylation, the T‐REx‐293‐TRPM4 cell model system was used to overexpress the channel in a tetracycline (Tet+)‐dependent manner (Armisén *et al*., 2011) and 9‐phenanthrol, a TRPM4 activity inhibitor (Grand *et al*., 2008), was used (DMSO was used as a vehicle control). As expected, overexpression of TRPM4 was accompanied by an increase in the total amount of β‐catenin and a decrease in its phosphorylated fraction, compared to noninduced (Tet−) cells (Fig. 5A,B). Significantly, the incubation with 9‐phenanthrol inhibited the effect of TRPM4 overexpression on the total β‐catenin levels and the phosphorylated fraction of this protein (Fig. 5A,B). Consistent with previous findings in LNCaP cells, TRPM4 overexpression also resulted in an increased inhibitory phosphorylation of GSK‐3β/Ser9 in Tet+ compared to Tet− cells (Fig. 5A, lane 1 *vs*. 3, Fig. 5B). Accordingly, a significant decrease in the Ser9 phosphorylation was observed upon incubation with 9‐phenanthrol (Fig. 5A, lanes 1 and 2, Fig. 5B). These results suggest that TRPM4 channel activity is required for GSK‐3β regulation and β‐catenin stabilization.

**Figure 6:**
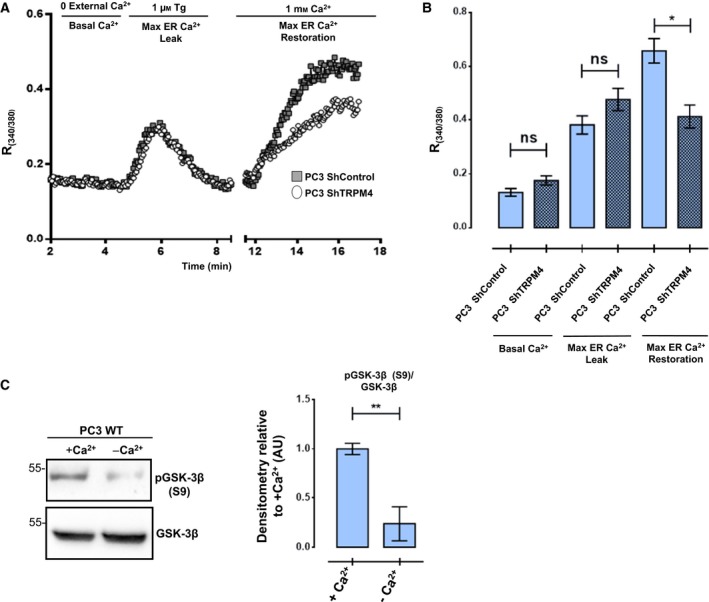
Modulation of calcium entry by TRPM4 in PC3 cells. (A, B) Intracellular calcium release and extracellular calcium entry were assessed through the incubation of thapsigargin (1 μm) and the restitution of extracellular calcium, respectively, using Fura‐2. A representative figure of one experiment is shown in (A). In (B), integrated data from six different experiments for PC3 ShControl and seven experiments for PC3 ShTRPM4 are shown. At least 10 cells were recorded in every experiment. TRPM4‐knockdown cells displayed a decrease in extracellular calcium entry compared to control cells. One‐way ANOVA, ^ns^ *P* > 0.05, *P ≤ 0.05. (C) PC3 WT cell lines were incubated with or without calcium and Ser9 phosphorylation of GSK‐3β was assessed with western blot assay. A representative western blot and the densitometries from three independent experiments are shown. Mean ± SEM are shown. *T‐*test with Welch correction, **P ≤ 0.01.

In several cell types, TRPM4 activity has been shown to modulate intracellular calcium concentration, through changes in membrane potential and in calcium electrochemical driving force (Fliegert *et al*., 2007; Gonzales *et al*., 2010; Launay *et al*., 2004). Given that calcium function is an intracellular second messenger (Berridge *et al*., 2003), we sought to determine whether TRPM4 expression modulates any component of calcium dynamics in PC3 cells. With the calcium‐specific probe Fura‐2, the calcium content in the ER was assessed by measuring ER calcium leak (in zero external calcium after SERCA inhibition with thapsigargin), and calcium entry was estimated by measuring cytosolic calcium upon external calcium replenishment after ER depletion. As shown in Fig. 6A,B, decreased TRPM4 channel expression in PC3 cells did not affect ER calcium leak. However, a significant decrease in calcium influx was observed when compared to control cells. To determine whether external calcium influx plays a role in the regulation of GSK‐3β activity, PC3 cells were incubated for 30 min in an extracellular medium free of calcium. A significant reduction in Ser9 inhibitory phosphorylation of GSK‐3β was observed (Fig. 6C), indicating that intracellular calcium concentration is important for this event.

### 3.3 Akt1 activation is regulated by Ca^2+^/CaM and TRPM4 in prostate cancer cells

**Figure 7:**
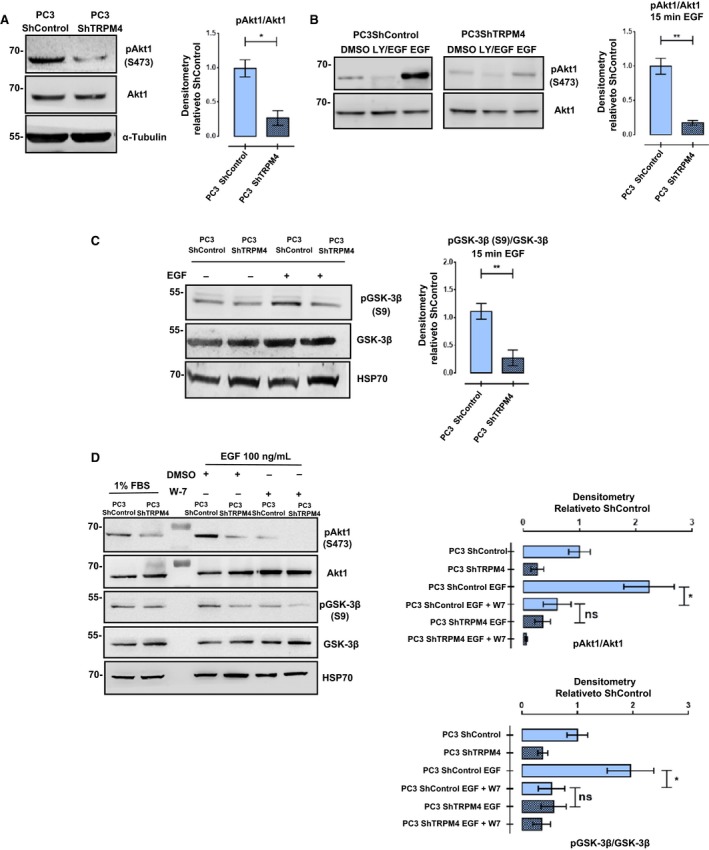
TRPM4 regulates the normal activation of Akt1 in PC3 cells. (A) PC3 ShControl shows an increased phosphorylation of Ser473 on AKT1 compared to PC3 ShTRPM4 under basal (nonstimulated) conditions. Representative western blots and their densitometries from three independent experiments (mean ± SEM) are shown. *T*‐test with Welch correction, *P ≤ 0.05. (B) PC3 ShControl and ShTRPM4 were incubated with EGF (100 ng·mL^−1^) for 15 min and phosphoactivation of AKT1 (Ser473) was evaluated. Incubation with LY294002 (20 μm) 1 h before EGF stimulus was used as a control. *T‐*test with Welch correction **P ≤ 0.01. (C) Downregulation of TRPM4 in PC3 cells impairs the inhibitory phosphorylation on GSK‐3β induced by EGF stimulation (100 ng·mL^−1^ per 15 min). *T‐*test with Welch correction, **P ≤ 0.01. (D) Inhibition of calmodulin in PC3 ShControl cells by W‐7 incubation (100 μm/30 min) before EGF incubation (100 ng·mL^−1^ per 15 min) resembles the effect of TRPM4 knockdown on Akt1 and GSK‐3β phosphorylation. Representative western blots and densitometries from three independent experiments are shown. Mean ± SEM are shown. One‐way ANOVA, ^ns^ *P* > 0.05; *P ≤ 0.05.

Akt1 kinase activation after EGFR stimulation is a process that involves Ca^2+^/CaM (Deb *et al*., 2004; Rokhlin *et al*., 2007) interaction in breast cancer cell models (Dong *et al*., 2007). After activation, Akt1 phosphorylates GSK‐3β at Ser9 and inhibits its function (Manning and Cantley, 2007). On the other hand, deregulation of Akt signaling is a common alteration in prostate cancer (Li *et al*., 2005). The status of Akt1 activity was measured by detecting its phosphorylation at specific residue Ser473 (Sarbassov *et al*., 2005). Under basal conditions and after EGF stimulation, Akt1 phosphorylation was lower in PC3 ShTRPM4 cells compared to control cells (Fig. 7A,B). Conversely, the overexpression of TRPM4 in LNCaP cells induced an increase in the Akt1 activating phosphorylation compared to WT and mock cells (Fig. S4), indicating a direct relationship between TRPM4 levels and Akt1 activation. Moreover, EGF‐induced GSK‐3β phosphorylation in Ser9 was also reduced in PC3 ShTRPM4 cells compared to control cells (Fig. 7C), which is consistent with the reduced Akt1 kinase activity in these cells. Finally, in order to explore the importance of EGF‐Ca^2+^/CaM signaling in both Akt1 and GSK‐3β phosphorylation, the effect of EGF stimulation was assessed in the presence of W‐7, a Ca^2+^/CaM inhibitor. PC3 ShControl cells preincubated with W‐7 showed a decrease in Akt1 and GSK‐3β phosphorylation levels after EGF stimulation, displaying phosphorylation levels similar to those detected in PC3 ShTRPM4 cells (Fig. 7D). Although a decrease in Akt1 phosphorylation was detected in PC3 ShTRPM4 cells incubated with W‐7, the inhibitor had no effect on GSK‐3β phosphorylation in these cells. These results highlight the importance of the TRPM4/Ca^2+^/Akt1 axis on the regulation of GSK‐3β activity and consequently the β‐catenin stability in prostate cancer cell lines.

## 4 Discussion

Several studies on prostate cancer have suggested that the expression of TRPM4 is a relevant event in the progression of this tumor (Holzmann *et al*., 2015; Schinke *et al*., 2014). Moreover, TRPM4 expression seems to have clinical relevance.

Berg *et al*. (2016) described a positive correlation between the overexpression of TRPM4 in prostate cancer samples and an increased risk of recurrence after radical prostatectomy. Schinke *et al*. (2014) showed that the TRPM4 gene is involved in the progression of the androgen‐independent growth stage, a late step in the progression of this tumor with no satisfactory treatment, indicating that this gene is an important candidate for study as a possible target for therapy in these patients.

TRPM4 could modulate a plethora of different signaling pathways given its intrinsic capacity to regulate the intracellular calcium concentration (Nilius and Vennekens, 2006), and could become an important player in different cellular processes, such as cell proliferation (Launay *et al*., 2004), migration (Holzmann *et al*., 2015; Shimizu *et al*., 2009), and apoptosis (Simon *et al*., 2010). Recently, Holzmann *et al*. (2015) described the role of TRPM4 in the regulation of store‐operated currents in prostate cancer cell lines and its potential impact on cellular migration. Using a siRNA against TRPM4, the primary human prostate epithelial cells (hPEC) and DU145 cells display an increase in SOCE, but no effect was observed in PC3 cells. In our work, PC3 ShTRPM4 cells display a decrease in SOCE (Fig. 6). This apparent discrepancy could be explained by several technical factors such as different cell culture conditions, extracellular bath solutions, and the use of a siRNA or shRNA against TRPM4 mRNA. The different approaches were used for knocking down TRPM4, which generates an acute or chronic reduction in TRPM4, and therefore, a distinct effect on gene expression could have an effect beyond the TRPM4 gene. Our group has previously shown that stable TRPM4 knockdown modifies the cellular phenotype, reversing the endothelial (Echeverría *et al*., 2015) or epithelial/mesenchymal transition (A. I. Sagredo, K. Marcelain & R. Armisén, unpublished data), suggesting new biological roles for TRPM4 expression beyond its function as a local calcium regulator. Intriguingly, Holzmann's group reported that the decrease in TRPM4 expression in DU145 and PC3 cell lines did not affect cell proliferation (Holzmann *et al*., 2015). Nevertheless, our data are consistent with previously published results in HeLa cells (Armisén *et al*., 2011) and with reduced levels of c‐Myc and cyclin D1 mRNA found after TRPM4 knockdown in our PC3 cell models.

We previously reported the effect of TRPM4 channel on cell proliferation through the regulation of the oncoprotein β‐catenin in HeLa cancer cells (Armisén *et al*., 2011), which promoted its stability and transcriptional function. This current work strongly supports a relationship between TRPM4 levels and β‐catenin stability and function in prostate cancer cell lines. Here, an increase in the activity of GSK‐3β in TRPM4‐knockdown cells is shown to correlate with a reduction in the total amount of β‐catenin, a change in its intracellular localization, and its function in the transcription of genes related to cell proliferation. Consistently, the role of TRPM4 in the regulation of β‐catenin signaling through GSK‐3β has been described in a colon cancer cell study (Major *et al*., 2008). Importantly, the analysis of 10 gene expression datasets from patients with prostate cancer and their controls shows that the most enriched pathway coexpressed with the TRPM4 gene is the Wnt signaling pathway, supporting our *in vitro* results and sustaining a relationship between the expression of this channel and the activity of this signaling pathway in prostate cancer (Fig. S5). Interestingly, we did not observe a significant increase in β‐catenin protein levels in PC3 ShControl and PC3 ShTRPM4 cells upon Wnt3a ligand stimulation, suggesting that the canonical pathway is already activated in these cells (Fig. S6). Moreover, these results suggest that the effect of TRPM4 over β‐catenin stability could be through a different molecular mechanism. Although TRPM4 and β‐catenin are in adhesion complexes (Cáceres *et al*., 2015; Valenta *et al*., 2012), we did not detect an interaction between these proteins (Fig. S7), suggesting a nonprotein–protein interaction effect of TRPM4 over β‐catenin activity.

While Wnt and Akt/PKB are the canonical regulators of GSK‐3β function, a number of reports have shown that calcium‐regulated proteins also participate in the modulation of GSK‐3β activity. GSK‐3β is phosphorylated *in vitro* by classical protein kinase C isoforms (Goode *et al*., 1992), and this phosphorylation results in GSK‐3β inactivation (Goode *et al*., 1992). It has also been shown that the inhibitory phosphorylation of GSK‐3β in serine 9 is reversed by protein phosphatases such as calcineurin (CaN) and PP2A (Kim *et al*., 2009). In addition, it has been shown that calpain, a calcium‐dependent intracellular protease (Medina and Wandosell, 2011), cleaves GSK‐3β, removing the GSK‐3β N‐terminal inhibitory domain with the net result of an increase in GSK‐3β activity (Goñi‐Oliver *et al*., 2007). Finally, the mechanism described in this work involves Ca^2+^/calmodulin (CaM), the principal Ca^2+^ sensor in eukaryotes (Hoeflich and Ikura, 2002), and EGF receptor signaling. It has been shown that Akt1 activation after EGFR signaling requires Ca^2+^/CaM binding to Akt1 (Dong *et al*., 2007). In this work, the activation of Akt1 under basal conditions is significantly reduced in TRPM4‐knockdown cells and correlates with a decrease in Ser9 GSK‐3β phosphorylation and β‐catenin signaling. Therefore, as the knockdown of TRPM4 channel is associated with a reduction in extracellular calcium influx, we propose that TRPM4 modulates the Ca^2+^/CaM signaling and indirectly regulates the activation of Akt1 affecting the downstream signaling events Ser9 GSK‐3β phosphorylation and β‐catenin stability. To support these results, we used the CaM inhibitor W‐7, before EGFR stimulation, and then detected the activation of Akt1 (pSer473) and pGSK‐3β (pSer9). Interestingly, the inhibition of calmodulin in PC3 ShControl cells resembles the results found for PC3 ShTRPM4 on Akt1 activity, suggesting a diminished activity of CaM in TRPM4‐knockdown cells. These results indicate a signaling axis composed of TRPM4‐Ca^2+^/CaM and EGFR‐Akt1. We tested the role of Akt1 as the main Ca^2+^‐regulated kinase on TRPM4 activity, evaluating GSK‐3β Ser9 phosphorylation postincubation with the drug TCN (Dieterle *et al*., 2009), a specific inhibitor of Akt (Fig. S8). We observed that the effect of EGFR stimulation on GSK‐3β phosphorylation was reduced in PC3 ShControl cells incubated with TCN, to levels similar to those for nonstimulated condition and PC3 ShTRPM4 cells. These results indicate that the main kinase responsible for the phosphorylation of GSK‐3β is Akt1 in our model. Nevertheless, further work will be needed to determine whether other calcium‐dependent kinases are involved in this process.

Finally, this work shows the involvement of TRPM4‐dependent calcium signaling in the regulation of β‐catenin and provides a framework to understand the contribution of a series of ion channels whose expression and/or function is altered in the tumor progression process (Farfariello *et al*., 2015; Flourakis and Prevarskaya, 2009; Prevarskaya *et al*., 2011).

## Author contributions

KM, RA, JCT, and AIS conceived and designed the project. AIS, EAS, CC, PB, REA, CB, and CE performed the experiments. RA, KM, EAS, OC, AS, FS, and JCT analyzed and interpreted the data. KM, RA, and AIS wrote the manuscript.

## Conflict of interest

The authors declare that they have no competing interests. EAS and RA are employees of Pfizer Chile.

## Supporting information


**Fig. S1.** TRPM4 expression in prostate cancer cell lines.

**Fig. S2.** Specificity of ShRNA used against TRPM4.

**Fig. S3.** No difference is observed in basal apoptosis levels of PC3 ShControl and TRPM4‐Knockdown cells.

**Fig. S4.** Overexpression of TRPM4 increases the activation of Akt1.

**Fig. S5.** TRPM4 coexpression signature across 10 prostatic cancer datasets.

**Fig. S6.** Wnt pathway activation in PC3 cells did not significantly increase the total β‐catenin protein levels.

**Fig. S7.** TRPM4 does not interact with β‐catenin.

**Fig. S8.** Akt1 is the main kinase responsible for GSK‐3β phosphorylation.

**Data S1.** Materials and methods.
