# Supplementary figures and images for: TRPM4 regulates Akt/GSK3‐β activity and enhances β‐catenin signaling and cell proliferation in prostate cancer cells
Source: Mol Oncol. 2017 Dec 30;12(2):151–65. doi: 10.1002/1878-0261.12100 (PMC5792731; doi:10.1002/1878-0261.12100)

**A**

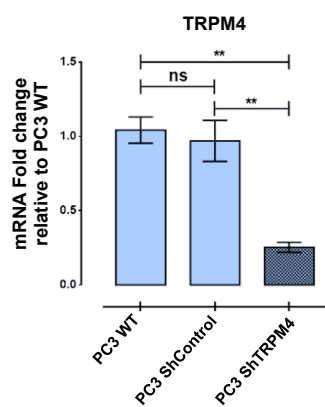

**B**

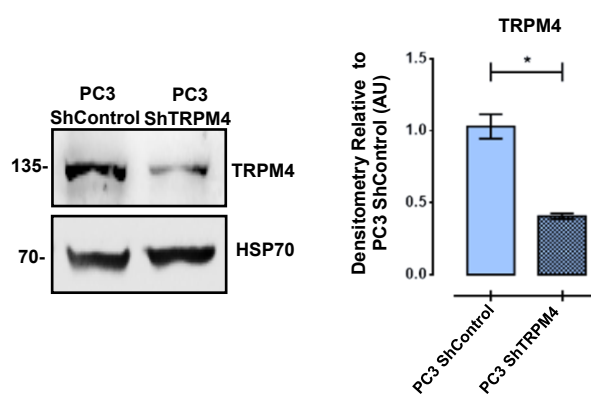

**C**

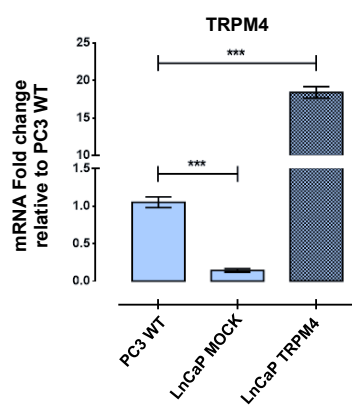

**D**

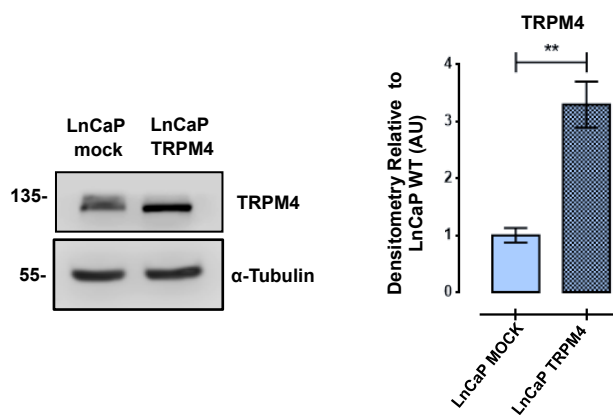

Supplement: Supplementary file 1 — Fig. S1. TRPM4 expression in prostate cancer cell lines. [file MOL2-12-151-s001.pdf]

**A**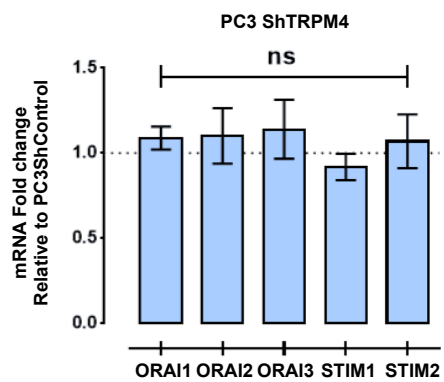

Supplement: Supplementary file 2 — Fig. S2. Specificity of ShRNA used against TRPM4. [file MOL2-12-151-s002.pdf]

**A**

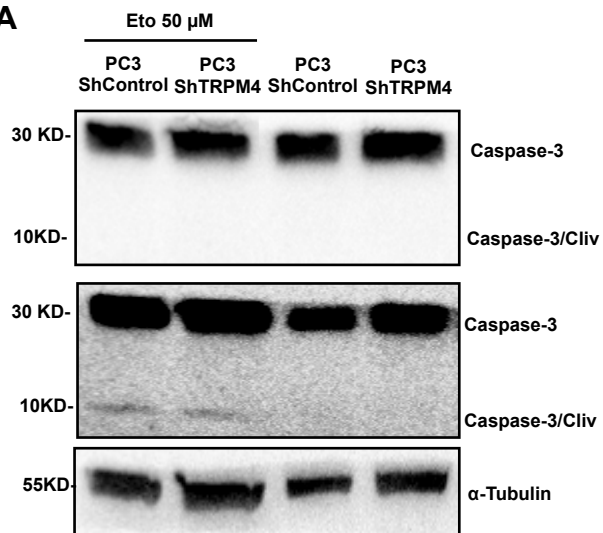

**B**

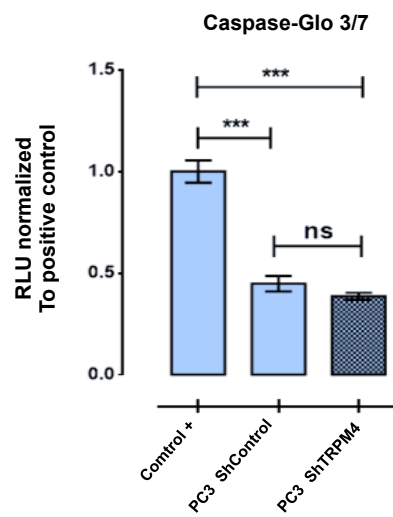

Supplement: Supplementary file 3 — Fig. S3. No difference is observed in basal apoptosis levels of PC3 ShControl and TRPM4‐Knockdown cells. [file MOL2-12-151-s003.pdf]

A

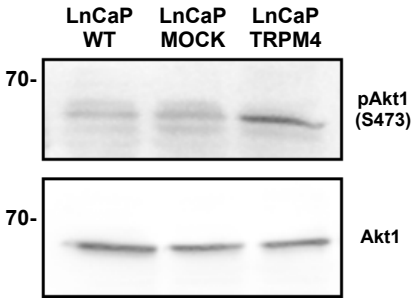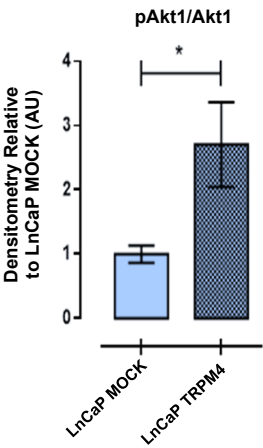

Supplement: Supplementary file 4 — Fig. S4. Overexpression of TRPM4 increases the activation of Akt1. [file MOL2-12-151-s004.pdf]

A

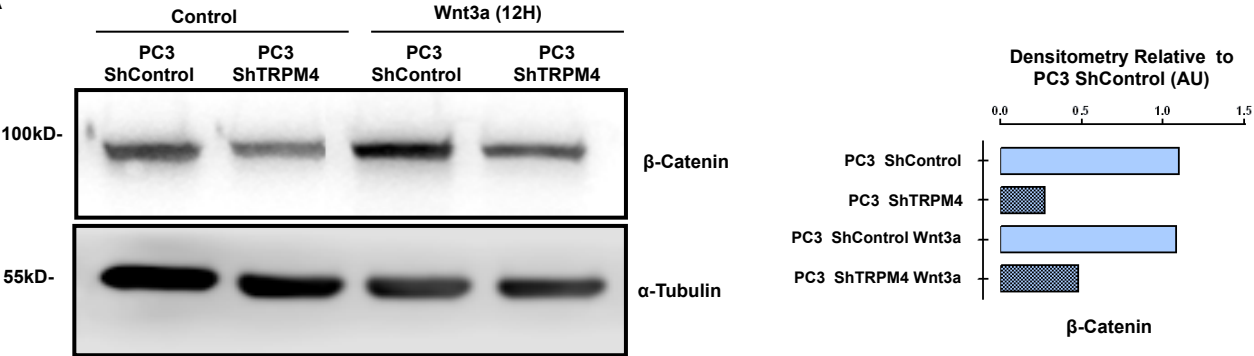

Supplement: Supplementary file 6 — Fig. S6. Wnt pathway activation in PC3 cells did not significantly increase the total β‐catenin protein levels. [file MOL2-12-151-s006.pdf]

Supp 7

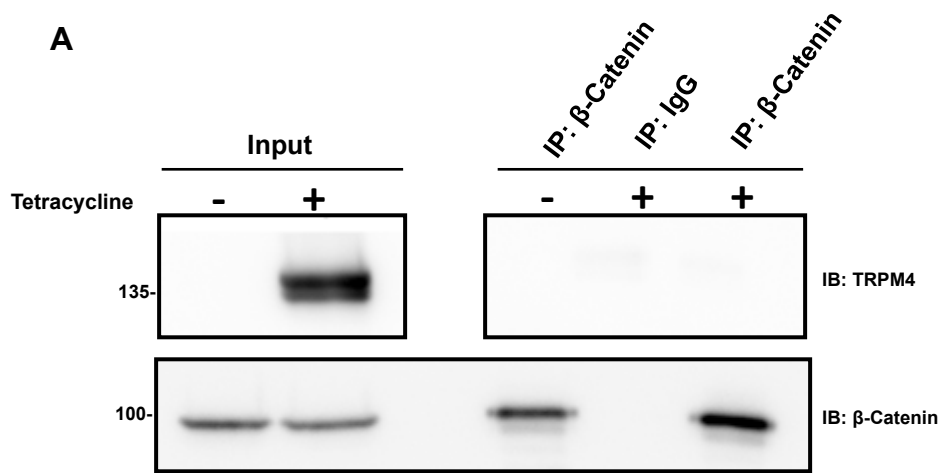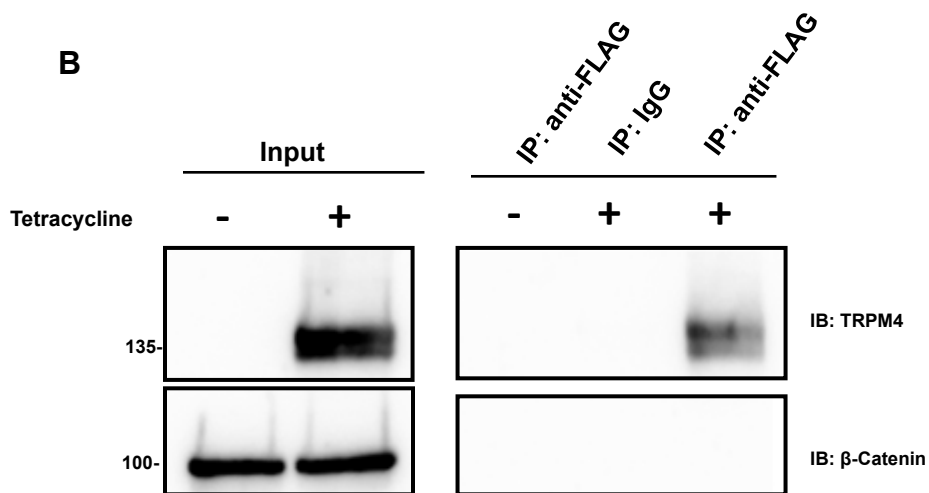

Supplement: Supplementary file 7 — Fig. S7. TRPM4 does not interact with β‐catenin. [file MOL2-12-151-s007.pdf]

A

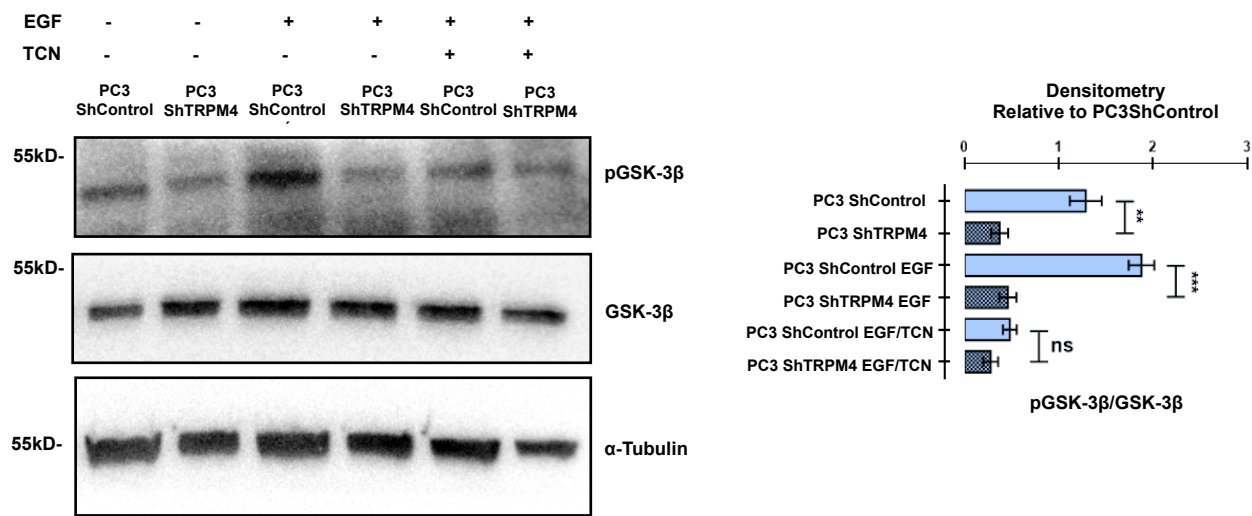

Supplement: Supplementary file 8 — Fig. S8. Akt1 is the main kinase responsible for GSK‐3β phosphorylation. [file MOL2-12-151-s008.pdf]
