# Supplementary material for: TRPM4 regulates Akt/GSK3‐β activity and enhances β‐catenin signaling and cell proliferation in prostate cancer cells
Source: Mol Oncol. 2017 Dec 30;12(2):151–65. doi: 10.1002/1878-0261.12100 (PMC5792731; doi:10.1002/1878-0261.12100)
Supplement: Supplementary file 5 — Fig. S5. TRPM4 coexpression signature across 10 prostatic cancer datasets. [file MOL2-12-151-s005.pdf]

Supp 5

GO Enrichment	Present in the dataset	Not present in the dataset
**Wnt signaling pathway**	8	2
**Gonadotropin releasing hormone receptor pathway**	6	4
**Inflammation mediated by chemokine and cytokine signaling pathway**	5	5
**Heterotrimeric G-protein signaling pathway-Gi alpha and Gs alpha mediated pathway**	1	9
**Angiogenesis**	1	9
**Integrin signalling pathway**	1	9
**PDGF signaling pathway**	1	9
**Huntington disease**	1	9

REFLIST (20000)	Enrichment Order	Present in the cluster	Expected number	Tendency
GSE3325	3	31	28.9	+
GSE6959	2	18	14.36	+
GSE8218	1	17	10.66	+
GSE12378	3	6	9.97	-
TABM 26	1	270	201.87	+
GSE17951	1	25	18.07	+
GSE21034	1	39	24.69	+
GSE26910	2	36	28.03	+
GSE29079	2	8	10.87	-
GSE6919	1	14	13.45	+
