# Supplementary material for: TRPM4 regulates Akt/GSK3‐β activity and enhances β‐catenin signaling and cell proliferation in prostate cancer cells
Source: Mol Oncol. 2017 Dec 30;12(2):151–65. doi: 10.1002/1878-0261.12100 (PMC5792731; doi:10.1002/1878-0261.12100)
Supplement: Supplementary file 9 — Data S1. Materials and methods. [file MOL2-12-151-s009.docx]

**Supplementary materials and methods.**

**1. Apoptosis assay.**

Detection of caspase-3/7 activation was made using the Caspase-Glo 3/7 Assay Systems (Promega), following manufacturer´s instructions (Promega). Measurements were done in triplicates. As a positive control, cells were exposed to sorbitol 300 mM for 8 hours. Luminescence was detected using the Cytation 3 Multi-Mode Reader (BioTek Instruments).

**2. Antibodies.**

Rabbit anti-caspase-3 (Cell Signaling, 9662) was used to detect total caspase-3 and its cleavage form using a western blot assay.

**3. Drugs and recombinant protein.**

For the activation of canonical Wnt Pathway, recombinant Human Wnt3a (R&D System, 5036-WN) was used (100 ng/mL/24 hours). The Akt inhibitor TCN Triciribine, TCN, (sc-200661) was used at a final concentration of 20 mM for 2 hours. Etoposide (TOCRIS, 1226) was used as an apoptosis positive control at a final concentration of 50 µM for 12 hours.

**4. Primers sequences (5’->3’) :**

These primers were used to analyze the ShRNA-TRPM4 off-target effect on SOCE genes.

ORAI1 (FwTTTATCGTCTTCGCCGTCCACT, RvTGGTCCTGTAAGCGGGCAAA),

ORAI2 (FwAGGAAGCACCAGACAGCCTTTT, Rv ACAAGATCGCTTGAACCTGGGA),

ORAI3 (TGGGACCTTCAGTGCTGACTTT, Rv CACTACACTCCAGCCTGGGAAA),

STIM1 (Fw CACAGTGAGAAGGCGACAGGAA, Rv ACTGCCTCGAAGCTGAGTTTCT),

STIM2 (Fw AGGATAGCAGTGCACGAACCTT, Rv AACCACATCCAATGCCTTGAGC)

**5. Immunoprecipitation assay.**

Immunopurification assays were performed according to *Cáceres et al 2015* (Cáceres et al., 2015). Briefly, TREx-293 TRPM4 cells were plated at 80% confluency on 60 mm dishes and TRPM4 expression was induced with 1µg/mL tetracycline for 24 h. Cells were solubilized in lysis buffer containing 0.5% v/v NP-40, 50 mM NaCl, 50 mM Tris/HCl (pH 8.0), 5 mM NaF, 1 mM phenylmethylsulfonyl fluoride and protease inhibitor cocktail for 30 min at 4 ˚C, followed by centrifugation at 12,000 x g for 10 min at 4 ˚C. The supernatants were incubated with 3µg of anti-FLAG (Sigma, F7425) and 1.5 µg of anti-β-catenin (BD Biosciences, 610154) overnight at 4 ˚C, followed by the addition of protein A sepharose beads (GE Amersham, Piscataway, NJ, USA) for 1 h at 4 ˚C. The beads were washed ten times in lysis buffer and immunopurified proteins were eluted by boiling in reduced SDS sample buffer (RSB).

**6. Analysis of TRPM4 coexpressed genes in human prostate cancer tumors.**

Ten public datasets of prostate cancer (GSE3325, GSE6919, GSE6956, GSE8218, GSE12378, GSE17951, GSE21034, GSE26910, GSE29079 and TABM26) were downloaded from GEO and/or ArrayExpress databases. These datasets contain the normalized gene expression values of prostatic cancer patients and their controls, including the expression values of TRPM4. We performed a *K-means* clustering (K=20, with 100 iterations) in Multiexperiment Viewer software (MeV 4.9) for all datasets. The gene cluster which contains the TRPM4 expression values was extracted and input into the Database for Annotation, Visualization and Integrated Discovery (DAVID, v6.7) using the PANTHER database to identify the pathway enrichment associated with the TRPM4 coexpression group.

**Supplementary figure legends.**

**Supplementary Figure 1. TRPM4 expression in prostate cancer cell lines.**

A and B. Lentiviral transduction of a specific ShRNA against TRPM4. PC3 cells were transduced with prepackaged lentivirus coding a ShRNA against TRPM4 or a ShRNA scramble as control. RT-qPCR and western blot were performed to confirm the knockdown of TRPM4. A) Relative mRNA expression of TRPM4. Mean +/- SEM from 3 independent experiments are shown. ns: p > 0.05, **p ≤ 0.01, *One way ANOVA.* B) Representative western blots and the densitometries of 3 independent experiments are shown. *p ≤ 0.05, *Student´s t-test*. C and D. TRPM4 overexpression in LNCaP cells. LNCaP WT cells were transfected with 2 µg of pCDNA4TO-TRPM4b or mock and 48 hours later the expression of TRPM4 was measured by qRT-PCR and western blot. C*)* Relative mRNA levels*.* Mean +/- SEM of 3 independent experiments are shown*.* ***p ≤ 0.001, *One way ANOVA,*. D) Representative western blots and the densitometry of 3 independent experiments are shown. **p ≤ 0.01, *Student´s T-test*.

**Supplementary figure 2. Specificity of ShRNA used against TRPM4.**

Possible off-target effect of ShRNA TRPM4 was evaluated with RT-qPCR. PC3 ShTRPM4 cells showed no differences in the mRNA expression levels of ORAI1, ORAI2, ORAI3, STIM1 and STIM2 relative to ShControl cells. All experiments were performed at least 3 times. Mean +/- SEM are shown. *Student´s T-tes*t, ns: nonsignificant differences.

**Supplementary Figure 3. No difference is observed in basal apoptosis levels of PC3 ShControl and TRPM4-Knockdown cells.**

A. Caspase-3 was detected in PC3 ShTRPM4 and ShControl cells by western blotting. No differences were detected in the expression of Caspase-3 and its cleavage form. Overnight incubation with Etoposide (Eto) 50 µM was used as a positive control. B. The caspase-Glo 3/7 assay (Promega) was used to measure basal apoptosis levels according to manufacturer's instructions. Nonsignificant differences of luminescence from these cell lines were detected in three independent experiments. Sorbitol at 300 mM for 8 hours was used as a positive control. At least 3 independent experiments were performed. Mean +/- SEM are shown. *One way ANOVA*, ns: p > 0.05, ***: p ≤ 0.001.

**Supplementary figure 4. Overexpression of TRPM4 increases the activation of Akt1.**

LNCaP WT cells were transfected with 2µg of TRPM4 expression plasmid or the empty vector (MOCK). 12 hours later, a western Blot of Akt1 and pAkt1 (Ser473) was performed. TRPM4 overexpression was correlated with an increase of Akt1 Ser473 phosphorylation while no differences in the total amount of Akt1 were detected. A representative western blot and the densitometry from 3 independent experiments (Mean +/- SEM) are shown. **p ≤ 0.05, T-test with Welch correction.*

**Supplementary Figure 5. TRPM4 coexpression signature across 10 prostatic cancer datasets.**

This figure summarizes the 8 most redundant GO (Gene Ontology) pathways associated with TRPM4 expression pattern in prostate cancer samples from 10 independent analyses. At the top is a summary table of the most redundant ontologies associated with a mRNA expression pattern of TRPM4. Below is a summary table with the order of enrichment of the Wnt signaling pathway in the 10 sets of prostate cancer data analyzed, indicating the enrichment order present for the Wnt signaling pathway, the number of Wnt addressable genes present in each dataset, the expected number of genes for Panther and the enrichment trend for each individual analysis.

**Supplementary figure 6. Wnt pathway activation in PC3 cells did not significantly increase the total β-catenin protein levels.**

PC3 ShControl and ShTRPM4 cells were incubated 12 hours with the Wnt3a ligand (100 ng/mL). After incubation, a western blot assay was performed to detect β-catenin in cell extracts. No differences were found in the total amount of β-catenin after Wnt3a incubation compared to basal conditions in all cellular models. N:2.

**Supplementary Figure 7. TRPM4 does not interact with β-catenin.**

Immunoprecipitation of endogenous β-catenin (A) and overexpressed TRPM4 (B) from a plasma membrane enriched protein fraction from tetracycline-treated (+) and untreated (-) T-REx-293 TRPM4 cells (see Materials and methods for details). Immunoblot of input and immunoprecipitation products (IP) from this assay are shown. Immunoprecipitation with mouse IgG and rabbit IgG were performed as controls for anti-β-catenin and anti-FLAG immunoprecipitations, respectively.

**Supplementary figure 8. Akt1 is the main kinase responsible for GSK-3β phosphorylation.**

A. Western Blot of GSK-3β/pGSK-3β was performed in PC3ShTRPM4 and ShControl. Cells were treated 2 hours with TCN (20mM) before the incubation of recombinant EGF (10 ng/mL), 15 minutes. Previous TCN exposure resembles the effect of TRPM4 knockdown in ShControl cells, suggesting that Akt is a main kinase involved in the phosphorylation of GSK-3β post-EGF stimuli. Representative western blots and their densitometries from 3 independent experiments. Mean +/- SEM are shown. *Multiple t-test comparisons using T-test with Welch correction,* ns: p > 0.05, **: p ≤ 0.01, ***: p ≤ 0.001.
